# In Vitro Evaluation of the Virucidal Activity of Ethanol Against Human Adenoviruses

**DOI:** 10.3390/v18101170

**Published:** 2026-10-21

**Authors:** Yuriko Matsumura, Hong Wu, Yoshihiko Fujioka, Youichi Suzuki, Atsuo Iwasawa, Takashi Nakano

**Affiliations:** 1 Department of Microbiology and Infection Control, Faculty of Medicine, Osaka Medical and Pharmaceutical University, 2-7 Daigaku-machi, Takatsuki 569-8686, Osaka, Japan; y-matsumura@thcu.ac.jp (Y.M.); hong.wu@ompu.ac.jp (H.W.); yoshihiko.fujioka@ompu.ac.jp (Y.F.); 2 Division of Infection Prevention and Control, Tokyo Healthcare University, 4-1-17 Higashi-Gotanda, Shinagawa-ku, Tokyo 141-8648, Japan; a-iwasawa@thcu.ac.jp; 3 Laboratory of Biosafety Research, Faculty of Medicine, Osaka Medical and Pharmaceutical University, 2-7 Daigaku-machi, Takatsuki 569-8686, Osaka, Japan; youichi.suzuki@ompu.ac.jp

**Keywords:** adenovirus, ethanol, inactivation, TEM

## Abstract

Ethanol has variable effectiveness against non-enveloped viruses; however, several reports suggest that it possesses virucidal activity against human adenoviruses (HAdVs). Ethanol concentrations between 45 w/w% and 95 w/w% showed sufficient activity against HAdV type 5 over a 30 s period, while concentration between 72.5 w/w% and 77 w/w% was effective against type 7 over 60 s period. Type 2 and 8 were less susceptible, however. In this study, we investigated the ability of different concentrations of ethanol to inactivate and change the structure of 19 HAdV types across six subgroups, using the virus infectivity titer for evaluation. After 1 min of ethanol treatment, HAdV types 5 and 8 were observed with transmission electron microscopy. The virucidal activity of disinfectant ethanol against HAdVs in subgroups A, B1 (except for type 21), and C was higher than that in subgroups B2, D, E, and F. We observed a ‘U-shaped’ fluctuation pattern where the reduction factor dropped at concentrations of 70–80% and then rose again at 90%. After treatment with 40% ethanol, TEM images showed no major changes in type 8 HAdVs, but organic matter was observed around the virus particles in type 5 HAdVs; after treatment with 66% ethanol, virus particles aggregated in both types. This suggests that HAdVs with a lower number of repeats in the shaft region exhibited higher ethanol resistance.

## 1. Introduction

Viral infections—spread via airborne transmission or contact with contaminated surfaces—pose a severe threat to the global human population. Since the novel coronavirus, SARS2-CoV-2 has precipitated the present outbreaks of COVID-19, and alcohol-based hand rubs have been used in daily hygiene practice all over the world to prevent contact transmission. Alcohol, especially ethanol, inactivates bacteria and enveloped viruses, with concentrations between 60% and 95% in hand rubs considered to be effective by the US Food and Drug Administration [1]. Ethanol is effective against many bacteria and inactivates enveloped viruses; however, it is generally ineffective against non-enveloped viruses [2,3]. Among the latter, human adenovirus (HAdV) causes mild infections in the upper and lower respiratory tracts, the pharynx, the conjunctiva, and the gastrointestinal tract [4,5,6]. Currently, 51 serotypes and over 70 genotypes have been described and classified within seven species (A to G) using bioinformatics analysis of complete genomic sequences, designated with consecutive numbers. Different types of HAdV display different tissue tropisms that correlate with clinical manifestations of infection [7,8,9,10]. For example, HAdV serotype 7 causes lower respiratory tract infections and severe pneumonia in children under 5 years of age, accounting for 3.5% to 11% of community-acquired pneumonia cases in children in China [11]; furthermore, the rate of severe disease is as high as 37% [12,13], and the mortality rate can reach 50% [4]. However, there are no effective antiviral drugs for adenovirus infections, and as such preventing the transmission of HAdV is crucial for infection control. Adenovirus virions possess an icosahedral capsid composed of 240 hexon trimers which account for approximately 60% of the total virion mass, with 12 vertices occupied by a pentamer of the penton protein and trimetric fiber proteins that extend from each penton base [4]. The fiber comprises three regions: the N-terminal ‘tail’, the central ‘shaft’, and the C-terminal ‘knob’. The number of repeats in the shaft moiety of a fiber protein depends on the serotype [14].

In a previous review of ethanol’s virucidal activity in hand disinfection [15], it was reported that concentrations between 45 and 95 w/w% showed sufficient activity against HAdV type 5 (subgroup C) over a 30 s period, concentrations between 72.5 and 77 w/w% were effective over a 60 s period against type 7 (subgroup B), and types 2 (subgroup C) and 8 (subgroup D) were less susceptible. It is thought that these differences are related to alcohol sensitivity, showing a protein-denaturing effect. As far as we are aware, no reports have investigated the relationship between HAdV type and ethanol’s virucidal activity in detail. Therefore, in this study, we focused on the inactivation of various HAdV types using different concentrations of ethanol, and additionally investigated structural changes after treatment using electron microscopy. Clinical observation studies on hand hygiene using alcohol-based antiseptics have reported that the median hand-rubbing time varied by facility, ranging from 17.4 s to a minimum of 7.3 s [16], where the average time taken in intensive care units was 7.6 s [17]. Notably, the minimum rubbing time recommended by numerous sources—a US study in 2022 [18], the S2k guideline on hand antisepsis and hygiene from Arbeitsgemeinschaft der Wissenschaftlichen Medizinischen Fachgesellschaften in 2023 [19], and the 3rd ICPIC alcohol-based handrub (ABHR) task force that built upon the World Health Organization (WHO) Guidelines on Hand Hygiene in Healthcare in 2024 [20]—to ensure hand hygiene is 15 s. In this study, we aimed to clarify the virucidal activity of ethanol; the contact time was set at 10 s (as the shortest feasible experimental contact time), and 1 min.

## 2. Materials and Methods

### 2.1. Materials

Rubbing alcohol was used for disinfection (FUJIFILM Wako Pure Chemical Corporation, Osaka, Japan). Different concentrations of ethanol (10 to 90 w/w%) were prepared (Guaranteed Reagent, FUJIFILM Wako Pure Chemical Corporation, Osaka, Japan) by mixing with sterile Milli Q water at room temperature before use.

### 2.2. Viruses and Cells

Viruses: HAdV types 1 (ATCC VR-1890), 2 (ATCC VR-846), 3 (ATCC VR-3), 4 (ATCC VR-1572), 5 (ATCC VR-5), 6 (ATCC VR-6), 7 (ATCC VR-7), 8 (ATCC VR-1932), 11 (ATCC VR-12), 12 (ATCC VR-863), 18 (ATCC VR-19), 19 (ATCC VR-1979), 21 (ATCC VR-256), 32 (ATCC VR-625), 37 (ATCC VR-929), 40 (ATCC VR-931), 41 (ATCC VR-930), and 53 (clinical isolate) were used. The titer of viral stock solution was 10^6~8^ TCID_50_/10 μL.

Cells: Adherent A549 cells (JCRB0076, human lung cancer origin) were used.

Media: 10% fetal bovine serum (FBS, NICHIREI BIOSCIENCES INC., Tokyo, Japan) and 1% penicillin–streptomycin–amphotericin B suspension (×100) (FUJIFILM Wako Pure Chemical Corporation) were added to Eagle’s MEM with non-essential amino acid (Shimadzu Diagnostics Corporation, Tokyo, Japan).

### 2.3. Antiviral Activity of Ethanol

A total of 100 μL of viral preparation was added to 900 μL of test solution, and the viral quantification assay was performed after 10 s and 1 min. Sterile Dulbecco’s phosphate-buffered saline (D-PBS(−)) served as a control. The viral test mixture was used to prepare a tenfold dilution series with the maintenance medium, in which 10 μL of each dilution was added to a 96-well microplate containing susceptible cells. The concentration of FBS in the viral preparation was kept at 1% during viral culture. After 2 h incubation for infection at 37 °C, 100 μL of medium was added and further incubated in 5% CO_2_ until quantification. Virucidal activity was determined after four to six days. Cytopathic effect (CPE) was observed and the 50% tissue culture infectious dose (TCID_50_) was calculated using the Behrens–Kärber method to determine (TCID_50_/10 μL). The *n* = 2 test was conducted on two different days, with a total of 4 independent repetitions. The detection limit of TCID_50_/10 μL is 0.67. The virucidal activity was calculated in terms of reduction factor (RF) by subtracting the logarithmic titers (logTCID_50_/10 μL) of the inactivated virus suspensions from that of the virus control, and was judged to occur when RF values were 4 or higher (RF ≥ 4).

### 2.4. Transmission Electron Microscopy (TEM) of Viral Particles

After HAdVs had been propagated in A549 cells for 6 days, each culture supernatant (10 mL) was centrifuged at 4 °C and 3000 rpm for 10 min, and then ultra-centrifuged at 4 °C and 150,000× *g* for 90 min. The residual pellet (50 μL) was used for morphological analysis.

After preparing 9 μL of the viral sample, 18 μL of ethanol solution was added, and the mixture was allowed to stand for 1 min before fixation with glutaraldehyde to a final concentration of 1%. The samples were applied to an ickel grid (400 mesh) supported by a carbon-coated collodion film, subjected to 1% (*w*/*v*) uranyl acetate for 20 s, and observed using TEM (HT7800 type, Hitachi, Tokyo, Japan). The accelerating voltage for the TEM observations was 80 kV. The grid was photographed with a CCD camera (XR-81) (Advanced Microscopy Techniques Corp (AMT), at a magnification of 25,000×. The pixel sizes of the micrographs taken under EM with the CCD camera were 322 ppi and 3296 × 2472.

## 3. Results

### 3.1. Virucidal Activity of Disinfectant Ethanol

**Figure 1:**
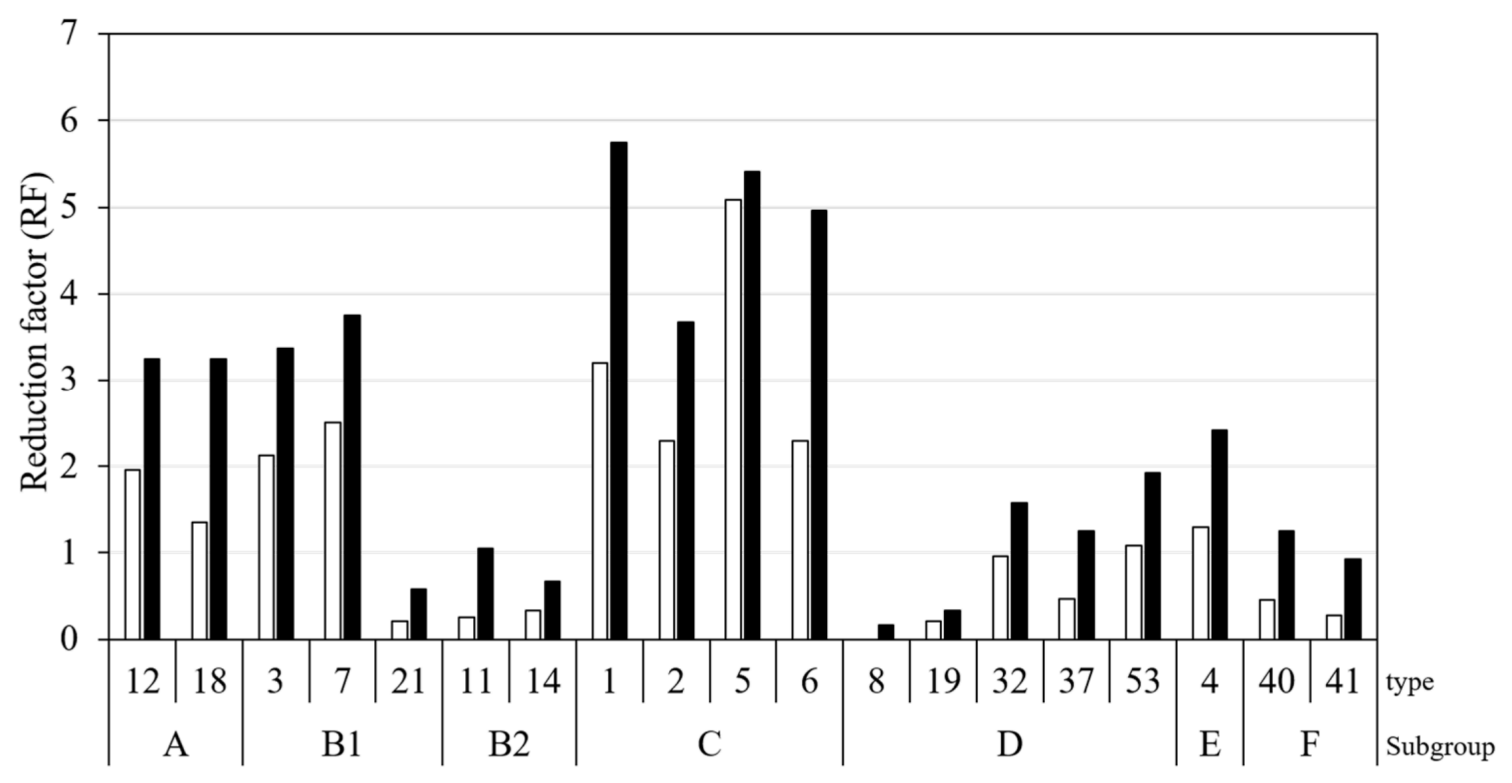
Virucidal activities against human adenoviruses after disinfection treatment with commercially available ethanol for 10 s (white bars) and 1 min (black bars) (*n* = 4).

The virucidal activities of commercially available ethanol for disinfection against various types of HAdV are shown in Figure 1. The RF values for contact times of 1 min were higher than for 10 s in all HAdVs, with viruses in subgroup A, B1 (except for type 21), and C showing higher RFs than those in subgroups B2, D, E, and F. For the HAdVs in subgroup D, the RF values for types 32, 37, and 53 were higher than those for types 8 and 19.

### 3.2. Virucidal Activity of Varying Ethanol Concentrations

**Figure 2:**
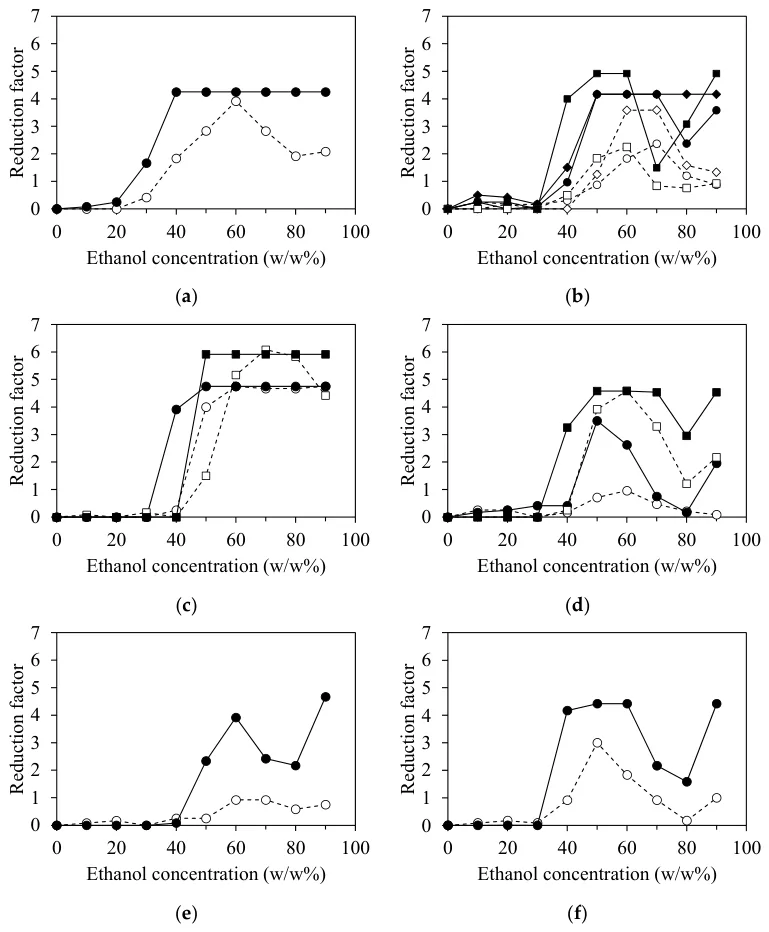
The influence of ethanol concentration on virucidal activity against HAdVs after treatment for 10 s (white symbols with dashed lines) and 1 min (black symbols with solid lines). (*n* = 4) (**a**) Subgroup A: type 12; (**b**) subgroup B: type 3 (○, ●), type 7 (◇, ◆), and type 14 (□, ■); (**c**) subgroup C: type 2 (○, ●) and type 5 (□, ■); (**d**) subgroup D: type 8 (○, ●) and type 37 (□, ■); (**e**) subgroup E: type 4; and (**f**) subgroup F: type 41.

The influence of varying ethanol concentrations of homemade ethanol (10 to 90 w/w%) on virucidal anti-HAdV activity is shown in Figure 2. After 10 s of contact, the RF value at ethanol concentrations below 30% was almost 0 for all HAdVs (open symbol). This phenomenon was also observed even after 1 min of contact, except for HAdV type 12 (subgroup A) (Figure 2a). When exposed to ethanol concentrations over 80% for 10 s, the RF value decreased compared to 70% ethanol for most types, but HAdVs in subgroups A and C showed lower values than those in other subgroups (Figure 2a,c). When exposed to ethanol for one minute, the RF values for HAdVs in subgroups A and C were 4 or more, and the virus titer was below the detection limit. For types 8 (subgroup D) (Figure 2d) and 4 (subgroup E), the RF values after 10 s of contact were lower than other HAdVs, regardless of ethanol concentration (Figure 2d,e, open symbol). While the RF values tended to increase with ethanol concentrations, they decreased at 70–80% concentrations and then rose again at 90%, showing a U-shaped change. This phenomenon was also observed in type 7 (subgroup B) and HAdVs not belonging to subgroup C.

### 3.3. Morphological Changes in HAdV Types 5 and 8 After Contact with Ethanol

**Figure 3:**
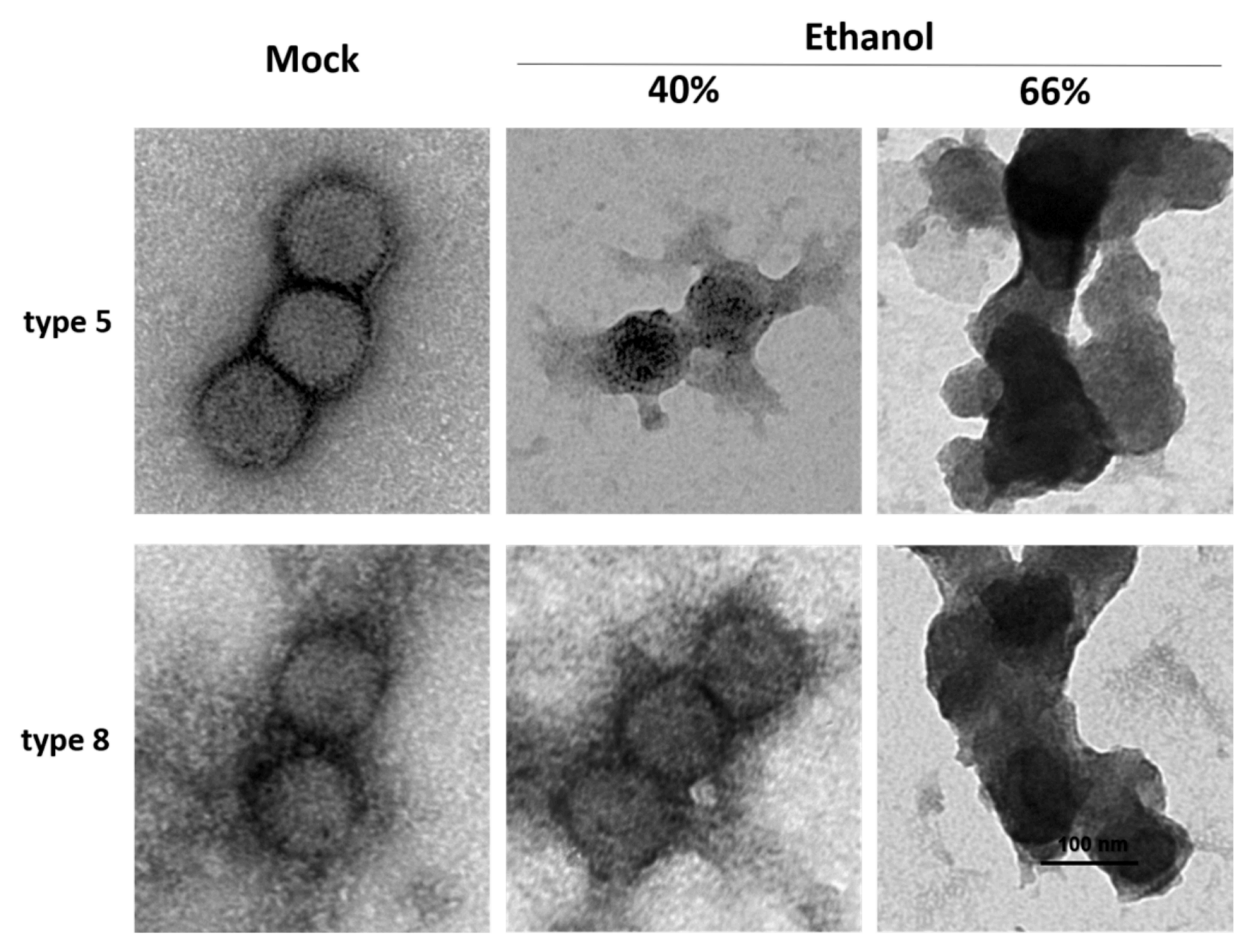
TEM images of HAdVs after treatment with 40% or 66% ethanol for 1 min.

TEM images of HAdV types 5 and 8 after contact with ethanol for 1 min showed the greatest difference in RF values compared to 10 s of contact with disinfectant ethanol (Figure 3). After contact with 40% ethanol, no major changes were observed in type 8, but organic matter was observed around the virus particles in type 5; after contact with 66% ethanol, virus particles aggregated in both types of HAdVs.

## 4. Discussion

**Figure 4:**
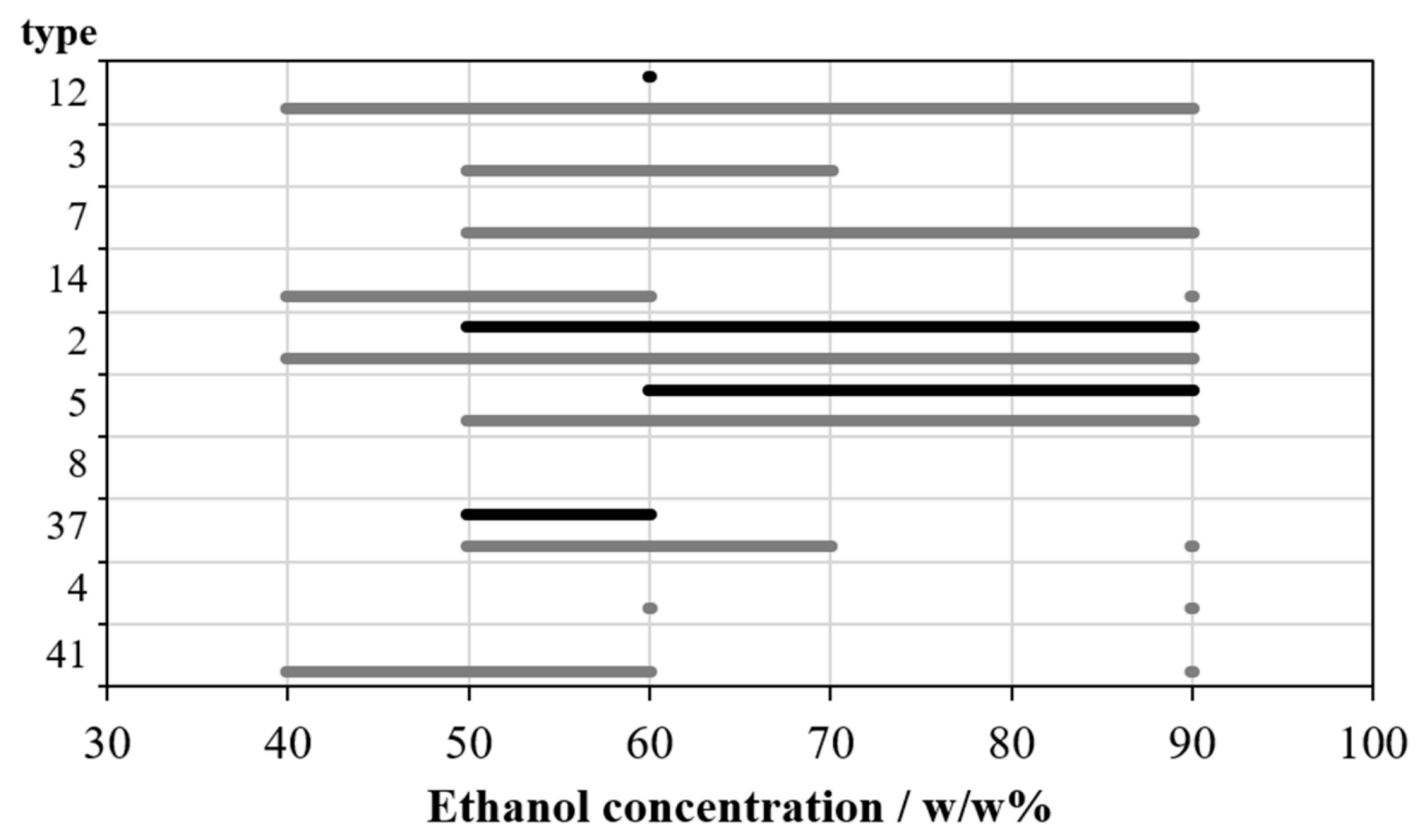
Effect of ethanol concentration on virucidal activity (RF ≥ 4) after treatment for 10 s (black bar) and 1 min (gray bar).

HAdVs are a family of double-stranded non-enveloped DNA viruses that may cause upper or lower respiratory tract infections, acute pharyngitis, pharyngoconjunctival fever, acute respiratory diseases, pneumonia, epidemic keratoconjunctivitis, acute hemorrhagic cystitis, and gastroenteritis depending on the types [4,5,6]. Over the past two decades, the components and organization of HAdV virions have been well studied extensively to determine their structural diversity and receptor-binding modes. The major coat protein, hexon, forms the icosahedron facets, and the capsomers at the vertices are formed by penton bases and fibers, playing a key role in the initial stage of infection [15]. As alcohol has a protein-denaturing effect, it is thought that its ability to inactivate adenoviruses is related. Although the recommended rubbing time when using alcohol-based hand antiseptic is at least 15 s [18,19,20], the minimum time spent on hand hygiene was reported to be 7.3 s in observational studies [16]. In this study, we set the shortest feasible experimental contact time with ethanol at 10 s and evaluated its virucidal activities against various types of HAdV. The virucidal activity of disinfectant ethanol on the 19 types of HAdV examined in this study (across six subgroups) was observed in half of all tested viruses. A total of eight HAdV types—six in subgroups A and C, and types 3 and 7 in subgroup B—showed a log_10_ titer reduction of three or above (Figure 1). Specifically, types 1 and 3 (subgroup C) showed this reduction after only 10 s of contact. In contrast, types 21, 11, and 14 (subgroup B), 8 and 9 (subgroup D), and (subgroup F) showed log_10_ titer reductions of approximately one or less after 1 min of contact, while types 32, 37, and 53 in subgroup D and type 4 in subgroup E showed log_10_ reductions of less than two. This means that disinfectant ethanol is only effective against certain types of HAdV, and not against subgroups B2, D, E, and F. HAdVs cause symptoms of epidemic conjunctivitis, whilst subgroup E comprises type 4, which has caused a moderate number of reported epidemic keratoconjunctivitis cased and acute respiratory diseases; subgroup F comprises HAdVs that are detected relatively frequently in acute gastroenteritis in infants and young children [6], which warrant particular attention in terms of infection control measures. Previous studies have shown that, for type 5, a contact time of 30 s with ethanol concentrations ranging from 45 to 70 w/w% is generally sufficient to achieve an adequate inactivation effect when the EN 14476 test virus was used; however, there are reports indicating that, with 77 w/w% ethanol, sufficient inactivation is only achieved after 1 min [21,22,23,24]. Type 2 exhibited virucidal activity when treated with 55–85 w/v% concentrations of ethanol after 2 min of contact [25,26]. There are also reports indicating that 72.5–77 w/w% ethanol was effective against type 7 within 1 min but was not effective against type 8 [27]. It has been reported that, against type 40, the log_10_ reduction value increases as the ethanol concentration and contact time increase; contact for 10 min at a concentration of 80% resulted in a log_10_ reduction of 2.33 or more, with virucidal activity also dependent on the ethanol concentration and contact time [28]. As testing methods vary between studies, we investigated virucidal effects under identical experimental conditions and using the same methods. The results are shown in Figure 4, in which samples showing an RF value of four or higher (RF ≥ 4) were judged to possess virucidal activity. The ethanol concentrations that demonstrated activity after 10 s of contact were 50–90 w/w% for type 2, 60–90 w/w% for type 5, 50–60 w/w% for type 37, and 60 w/w% for type 12. On the other hand, even after 1 min of contact, no virucidal activity was observed for type 8, whilst type 4 showed virucidal activity only within the narrow concentration range of 60–90 w/w%. Types 3 and 37 exhibited virucidal activity at 50–60 w/w%; types 5 and 7 over a wider concentration range of 40–90 w/w%; and types 12 and 2 showed the widest concentration range of 40–90 w/w%. This study has clear limitations that must be addressed before clinical application. We evaluated virucidal activity in vitro, as our cell culture system using A549 cells is clearly unnatural, and 1 min of exposure is a very long duration of exposure compared to general durations on clinical sites. The amount of biological materials (blood, feces, mucus, sputum, urine, etc.) coating such viruses in real-world exposure situations is very variable and was not explored/simulated in this study. In ward situations, the adenovirus type will not be immediately clear on patient presentation, so it will be ambiguous whether an alcohol-sensitive or -resistant type is present.

Focusing on the structure of the HAdV virion, the capsid consists of an icosahedral shell formed by hexon trimers, 12 pentameric penton capsomers at each vertex, and 12 fibers extending from the penton base, each of which comprises three regions: the N-terminal tail, the central shaft, and the C-terminal knob [4,14]. The rod-like shaft comprises a β-sheet consisting of a repeating structure limited to a maximum of 14 amino acid residues, and the number of repetitions varies between subgroups. In subgroup A, types 12 and 18 contain 22 [29] and 22.5 [29,30] repetitions, respectively; subgroup B contains six [29]; subgroup C contains 21–22, with type 6 alone having 18 repeats [29]; subgroup D contains 7.5 [29,31]; and type 4 in subgroup E contains 22 [29]. Subgroup F has a long region of 21–22 repeats and a short region of 12 repeats [32]. It was shown that types with a lower number of β-sheet repeats exhibited lower RF values as shown in Figure 1, and these HAdVs tended to demonstrate resistance to ethanol. These results support previous reports that significant differences in susceptibility to disinfectants such as ethanol are due to factors such as the physical density of the protein loops exposed on the surface of HAdVs [15]. It was hypothesized that the reason why subgroup F, which possesses both long and short regions, exhibits greater alcohol tolerance than subgroups A and C, which have a higher number of repeats, is attributable to the short regions in subgroup F. In this study, we did not analyze the structure for each serotype, so these findings remain speculative; however, our results suggest that types with fewer shaft repeats exhibit higher ethanol resistance. We are focusing on the structural changes in types 5 and 8, which showed the greatest differences in RF values following 10 s of contact with disinfectant ethanol. In the TEM images taken one minute after contact (Figure 3), contact with 66% ethanol resulted in both organic matter leakage and virus particle aggregation in both types 5 and 8. In contrast, following contact with 40% ethanol, organic matter leakage was observed around the type 5 virus particles, whereas no such leakage was observed in type 8, which exhibited a similar appearance to the mock control. It has been reported that when adenovirus type 5 is treated with 70% ethanol at pH 4, the penton bases and fibers detach from the capsid, leading to capsid disintegration [33]. It is therefore presumed that the leakage of organic matter observed in this study consists of these detached penton bases and fibers. The shaft of type 5 consists of 22 repeats and is approximately 37 nm in length [34], whilst that of type 8 consists of eight repeats and is approximately 11–12 nm in length [29,35]. It is thought that this difference in fiber structure is one of the factors contributing to the variation in organic leaching between the two types upon contact with 40% ethanol. Hexon proteins contain two pedestal regions and four loops, exhibiting a very high degree of amino acid sequence homology even between types 5 and 8, and the three-dimensional folding that forms the trimer (the ‘double jelly-roll’ structure) is almost identical [36]. In contrast, the loop region of type 5 consists of amino acids 851–952, and the surface of the HVRs—particularly HVR7—exhibits a structure in which highly hydrophilic amino acids are exposed [37]. Conversely, in type 8, the HVR loop structure features clusters of highly positively charged and hydrophobic amino acids exposed in a patch-like pattern; HVR1 to HVR7 are three-dimensionally folded, forming specific molecular grooves on the sides of the tower, and the structure is stabilized by hydrogen bonds and salt bridges [38,39]. From the TME images shown in Figure 3, it is difficult to identify the hexons as the viral particles were obscured by the organic matter leached out in 66% ethanol; however, following exposure to 40% ethanol, structures presumed to be hexons were observed in both types. Consequently, taking hexon proteins into account, it is difficult to determine whether ethanol sensitivity differs between types. Our finding that ethanol sensitivity varies depending on the type of HAdV supports the view that this is due to structural differences.

## 5. Conclusions

Of the 19 types of HAdVs tested, disinfectant ethanol showed no effect on subgroups B2 and D, while E and F showed resistance to ethanol even after one minute of contact; however, for the six types belonging to subgroups A and C, and types 3 and 7 in subgroup B, contact for 10 s resulted in a virucidal effect of RF3 or higher. The TEM images suggest that HAdVs with a lower number of repeats in the shaft region exhibit higher ethanol resistance.

## Data Availability

The data presented in this study are available in the article.
